# Revealing the Three‐Dimensional Complexity of Facial Anatomy Through Micro‐Computed Tomography

**DOI:** 10.1111/jocd.70379

**Published:** 2025-08-08

**Authors:** Kyu‐Ho Yi, Jovian Wan, Jong Keun Song, Arthur Swift, Benjamin Ascher

**Affiliations:** ^1^ Division in Anatomy and Developmental Biology, Department of Oral Biology, Human Identification Research Institute BK21FOUR Project, Yonsei University College of Dentistry Seoul Korea; ^2^ You and I Clinic Seoul Korea; ^3^ Medical Research Inc. Wonju Korea; ^4^ Pixelab Plastic Surgery Clinic Seoul Korea; ^5^ Swift Beauty Clinic Toronto Ontario Canada; ^6^ SIBUS Inc. Paris France

**Keywords:** aesthetic injection safety, facial anatomy, micro‐CT, SIBUS, ultrasound

## Abstract

**Background:**

Micro‐computed tomography (micro‐CT) offers exceptional three‐dimensional resolution for studying facial anatomy, capturing intricate structural relationships with isotropic resolutions as fine as 4–10 μm. However, its use is limited to ex vivo specimens, precluding real‐time or functional evaluation.

**Objective:**

To highlight the complementary value of high‐frequency ultrasound in facial anatomical assessment and procedural guidance, particularly in the clinical context.

**Methods:**

We discuss the limitations of micro‐CT for dynamic applications and explore the clinical advantages of ultrasound, including Doppler capabilities and real‐time observation of soft tissue structures.

**Results:**

High‐frequency ultrasound (30–70 μm resolution) enables in vivo, dynamic imaging of vascular flow, muscle activity, and filler placement. It is non‐invasive, repeatable, and applicable at the point of care. Finger‐mounted devices, such as those used in Safe Injection By Ultrasound (SIBUS) protocols, provide sufficient resolution for guiding aesthetic procedures involving superficial facial anatomy. Doppler integration further enhances procedural safety.

**Conclusion:**

While micro‐CT remains ideal for high‐resolution anatomical research and educational modeling, ultrasound uniquely enables functional, real‐time assessment essential for safe clinical practice. Together, these modalities serve complementary roles in advancing both anatomical understanding and patient care in aesthetic medicine.

## 1 Introduction

The human face constitutes one of the most architecturally complex regions of human anatomy, where subtle variations in spatial relationships carry profound functional consequences. For centuries, anatomical understanding of this region has been constrained by inherent limitations in available visualization techniques. Traditional dissection methods, whilst invaluable for gross anatomical study, inevitably disrupt the precise three‐dimensional relationships they seek to demonstrate [1, 2]. Histological techniques provide excellent microscopic detail but sacrifice the essential macroscopic context required for understanding functional integration. Even modern virtual anatomy platforms, despite their purported three‐dimensional capabilities, frequently rely on simplified models that inadequately represent the true complexity of anatomical arrangements.

These methodological limitations are particularly problematic when studying structures exhibiting significant individual variation. The facial nerve, for instance, demonstrates considerable natural variation in its branching patterns between individuals [3]. Similarly, facial muscles frequently deviate from standard anatomical descriptions in their origins, insertions, and layering patterns [4, 5]. While discrepancies between textbook anatomy and actual specimens are well documented, contemporary teaching methods often fail to adequately convey this inherent anatomical variability [6].

Micro‐computed tomography (micro‐CT) offers a transformative approach to studying facial anatomy, addressing many traditional limitations while providing unique advantages over other imaging modalities. Modern micro‐CT systems achieve isotropic resolutions as fine as 4 μm, allowing detailed three‐dimensional examination of nerve fascicles, capillary networks, and connective tissue layers in their natural, undisturbed state. Crucially, this bridges the scale gap between macroscopic and microscopic anatomy [7]. Unlike magnetic resonance imaging, which excels in soft tissue differentiation but lacks sufficient resolution for fine structural detail, or ultrasound, which provides real‐time imaging but suffers from limited penetration depth, micro‐CT uniquely combines exceptional resolution with comprehensive volumetric visualization. This positions micro‐CT as an ideal tool for comprehensive facial anatomical investigation.

The application of micro‐CT technology in facial anatomy research has yielded significant insights, as visually demonstrated in Videos S1 and S2. These reconstructions reveal intricate spatial relationships and musculature attachment patterns, providing crucial anatomical context for surgical and aesthetic procedures. Recent micro‐CT studies have documented consistent patterns in facial nerve branching architecture, identified variations in regional vascular supply, and elucidated the three‐dimensional organization of fascial layer systems [7, 8]. Such findings substantially enhance our foundational understanding.

Micro‐CT imaging for soft tissue visualization typically employs high‐resolution scanners with optimized parameters. Standard protocols utilize X‐ray energies of 80–100 kV and currents of 100–200 μA, achieving voxel sizes of 4–10 μm. Specimen integrity is maintained during scanning, often through immersion in physiological saline, to preserve tissue architecture and prevent dehydration artifacts. Acquisition generates large datasets (typically 2000–4000 projection images per specimen over 2–4 h). Contrast‐enhanced techniques, frequently employing iodine‐based agents like Lugol's solution (1%–5% concentration) with 24–48 h incubation periods, significantly improve soft tissue differentiation, enabling clear delineation of neural, vascular, and fascial structures. Image reconstruction utilizes filtered back‐projection algorithms incorporating beam‐hardening corrections. Subsequent three‐dimensional rendering, segmentation, and quantitative analysis via specialized software facilitate comprehensive visualization of anatomical relationships and spatially accurate morphometric measurements. This integrated methodological approach underpins the technology's capability to advance our comprehension beyond schematic representations towards a comprehensive, scientifically validated three‐dimensional reality, paving the way for enhanced anatomical education and refined clinical interventions.

## 2 Innovative Ideas

The fundamental innovation of micro‐CT lies in its capacity to transcend the traditional scale limitations that have long constrained anatomical investigation. Whilst light microscopy excels at revealing cellular and subcellular detail but sacrifices macroscopic context, and gross dissection preserves anatomical relationships whilst obscuring fine structural detail, micro‐CT successfully bridges these domains. Contemporary systems achieve resolutions of 4–10 μm. This enables visualization of individual nerve fascicles and small vascular structures whilst maintaining the undisturbed three‐dimensional arrangement of entire anatomical regions.

This capability proves invaluable for elucidating the facial nerve's complex branching architecture. Recent micro‐CT applications have revealed functional pathway segregation within the main nerve trunk at surprisingly proximal levels, challenging established assumptions regarding random fiber distribution. The technology demonstrates consistent spatial relationships between the nerve and adjacent structures, such as the parotid ductal system, while simultaneously documenting the considerable individual variation inherent in facial neuroanatomy. These insights provide a more accurate foundation for understanding facial neurological relationships, carrying significant implications for both anatomical education and clinical training.

**FIGURE 1:**
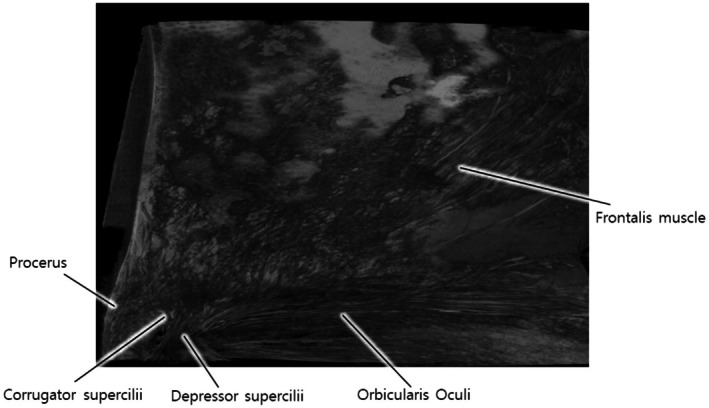
High‐resolution micro‐computed tomography (micro‐CT) reconstruction of the upper face demonstrating the three‐dimensional architecture of superficial and deep muscle fibers. Fine muscular fascicles, including portions of the frontalis, orbicularis oculi, and corrugator supercilii muscles, are clearly visualized in situ without disruption of spatial relationships, highlighting the utility of micro‐CT for detailed anatomical analysis of facial musculature.

Micro‐CT similarly transforms our understanding of muscular anatomy (Videos S1 and S2). The technology facilitates precise three‐dimensional mapping of muscle origins and insertions, frequently revealing substantial discrepancies with conventional anatomical descriptions in standard texts. The complex interlacing patterns of mimetic muscles, notoriously difficult to appreciate through traditional dissection, become clearly discernible in high‐resolution three‐dimensional reconstructions (Figure 1). These refinements substantially advance our comprehension of facial expression mechanics and provide a more accurate anatomical basis for interventions ranging from reconstructive surgery to therapeutic neuromodulation.

Vascular anatomy likewise benefits from micro‐CT's exceptional capabilities. The technology effectively maps intricate collateral networks essential for tissue viability while identifying anatomical variations that may predispose individuals to procedural complications. Such detailed vascular characterization proves critical for understanding flap physiology and anticipating potential sites of anatomical vulnerability during aesthetic and reconstructive procedures.

## 3 Discussion

Micro‐CT provides anatomical insights with significant implications for education and practice. Within academic settings, this methodology enables a paradigm shift from schematic representations towards evidence‐based three‐dimensional anatomical models. Such models more effectively accommodate natural anatomical variations encountered clinically while providing students with accurate comprehension of complex spatial relationships. Crucially, the non‐destructive nature of micro‐CT facilitates the creation of comprehensive digital anatomical atlases, preserving rare specimens for perpetual study and analysis.

For clinical training, micro‐CT datasets substantially enhance surgical simulators and virtual reality platforms through anatomically faithful representations derived from actual specimens rather than artistic interpretations. The technology's quantitative capabilities support precise documentation of anatomical parameters, thereby informing evidence‐based procedural guidelines and safety protocols, particularly relevant for minimally invasive techniques where subtle spatial relationships determine success.

Current research applications include systematic mapping of safe surgical planes, comprehensive identification of clinically significant anatomical variations, and development of enhanced educational models [7, 9, 10, 11]. The capacity for non‐destructive imaging further permits repeated examination of specimens under varying parameters, enabling multi‐modal analysis.

Implementation within medical curricula necessitates strategic consideration of technological infrastructure and pedagogical integration. This encompasses developing interactive three‐dimensional visualization platforms, virtual dissection tools, and accessible digital anatomical libraries compatible with standard educational technologies. Successful adoption further requires standardized annotation systems, quality assurance protocols, and assessment tools leveraging three‐dimensional understanding. Collaborative platforms could democratize access to rare specimens across institutions, reducing reliance on physical cadaveric resources.

Significant challenges nevertheless constrain broader adoption. Substantial equipment costs (£100 000–500 000), specialized technical expertise, and computational demands for processing high‐resolution datasets present formidable barriers, particularly for smaller institutions. Technical limitations include inherent soft tissue contrast constraints (necessitating contrast agents), artifact susceptibility in mineralized tissues, and specimen preparation complexities. Data interpretation requires niche expertise, whilst translating anatomical findings into clinical relevance remains technically demanding. Furthermore, ex vivo imaging limits direct clinical applicability, and functional correlation requires additional validation. Substantial data storage needs and specialized visualization software compound implementation hurdles.

Addressing these challenges mandates standardized protocols for specimen preparation, imaging parameters, and analysis. Validation frameworks must correlate micro‐CT findings with established knowledge while documenting clinically significant variations. This necessitates reference databases, quality metrics, and inter‐institutional collaboration. Standardization efforts should prioritize consensus imaging guidelines, uniform annotation systems, and accessible data‐sharing frameworks, requiring coordination between academia, industry, and regulators. While micro‑CT provides unparalleled three‑dimensional resolution, it remains inherently limited to ex vivo specimens, thereby precluding real‑time or functional assessment. In contrast, high‑frequency ultrasound offers a valuable complementary modality for facial anatomical study. Ultrasound enables in vivo, dynamic, and simultaneous observation of soft tissue structures, allowing clinicians to evaluate vascular flow, muscle contraction, and filler placement in real time. Although its spatial resolution (typically 30–70 μm with high‑frequency probes) is lower than that of micro‑CT, ultrasound possesses the unique advantage of non‑invasiveness, repeatability, and direct clinical applicability. The ability to integrate Doppler imaging further enhances vascular mapping and procedural safety during minimally invasive interventions. Finger mounted ( Safe Injection By Ultrasound (SIBUS)) ultrasound devices have proven adequate for imaging superficial facial structures, facilitating real‐time, point‐of‐care guidance. Thus, while micro‑CT excels in capturing static microstructural detail for research and educational purposes, ultrasound provides essential functional and safety information in the clinical setting, making the two modalities complementary rather than mutually exclusive

Future developments should prioritize standardized facial imaging protocols, comprehensive normative variation databases, and clinical outcome correlations. Integrating micro‐CT data with complementary modalities (e.g., magnetic resonance imaging) promises a more holistic anatomical understanding. Developing user‐friendly visualization platforms remains essential for wider educational adoption. Emerging artificial intelligence applications offer transformative potential through automated segmentation, pattern recognition, and educational content generation, potentially reducing analytical expertise barriers while enhancing resource accessibility. Collectively, these advances could ultimately transform micro‐CT from a specialized research tool into a cornerstone of evidence‐based anatomical education and clinical practice.

## 4 Conclusion

Micro‐CT imaging enables detailed visualization of facial anatomy by preserving complex three‐dimensional relationships at microscopic resolution. As illustrated in Videos S1 and S2, this technology captures both consistent anatomical patterns and significant individual variations in neural, vascular, and musculofascial organization that are inaccessible to conventional techniques. Although currently confined to research applications, micro‐CT has significant potential to enhance anatomical education, refine surgical approaches, and improve outcomes in facial interventions.

## Author Contributions

Kyu‐Ho Yi, Jovian Wan, Jong‐Keun Song, Arthur Swift, Benjamin Ascher: conceptualization. Kyu‐Ho Yi, Jovian Wan: writing – original draft preparation. Kyu‐Ho Yi, Jovian Wan, Arthur Swift, Benjamin Ascher: writing – review and editing, Kyu‐Ho Yi, Jovian Wan: visualization. Kyu‐Ho Yi: supervision. All authors have reviewed and approved the article for submission.

## Consent

Informed consent was obtained from all participants, with full disclosure of the study's purpose, risks, and confidentiality.

## Conflicts of Interest

The authors declare no conflicts of interest.

## Supporting information


**Video S1:** Three‐dimensional micro‐CT reconstruction of facial musculature demonstrating muscle architecture, attachment points, and intermuscular connections. Dynamic rotation reveals spatial relationships inaccessible through gross dissection (Scale: 1 mm increments).

**Video S2:** Contrast‐enhanced micro‐CT visualization of facial anatomy differentiating tissue interfaces. The technique demonstrates neurovascular pathways and fascial planes within undisturbed anatomical context (Scale bar: 500 μm).

## Data Availability

Data sharing is not applicable to this article as no new data were created or analyzed in this study.
